# Metformin use and improved response to therapy in rectal cancer

**DOI:** 10.1002/cam4.54

**Published:** 2013-02-03

**Authors:** Heath D. Skinner, Christopher H. Crane, Christopher R. Garrett, Cathy Eng, George J. Chang, John M. Skibber, Miguel A. Rodriguez-Bigas, Patrick Kelly, Vlad C. Sandulache, Marc E. Delclos, Sunil Krishnan, Prajnan Das

**Affiliations:** 1 Department of Radiation Oncology, The University of Texas MD Anderson Cancer CenterHouston, Texas, 77030; 2 Department of Gastrointestinal Medical Oncology, The University of Texas MD Anderson Cancer CenterHouston, Texas, 77030; 3 Department of Surgical Oncology, The University of Texas MD Anderson Cancer CenterHouston, Texas, 77030; 4 Department of Head and Neck Surgery, The University of Texas MD Anderson Cancer CenterHouston, Texas, 77030; 5 Bobby R. Alford Department of Otolaryngology-Head and Neck Surgery, Baylor College of MedicineHouston, Texas, 77030

**Keywords:** Chemotherapy, metformin, radiation, rectal cancer

## Abstract

Locally advanced rectal cancer is commonly treated with chemoradiation prior to total mesorectal excision (TME). Studies suggest that metformin may be an effective chemopreventive agent in this disease as well as a possible adjunct to current therapy. In this study, we examined the effect of metformin use on pathologic complete response (pCR) rates and outcomes in rectal cancer. The charts of 482 patients with locally advanced rectal adenocarcinoma treated from 1996 to 2009 with chemoradiation and TME were reviewed. Median radiation dose was 50.4 Gy (range 19.8–63). Nearly, all patients were treated with concurrent 5-fluorouracil-based chemotherapy (98%) followed by adjuvant chemotherapy (81.3%). Patients were categorized as nondiabetic (422), diabetic not taking metformin (40), or diabetic taking metformin (20). No significant differences between groups were found in clinical tumor classification, nodal classification, tumor distance from the anal verge or circumferential extent, pretreatment carcinoembryonic antigen level, or pathologic differentiation. pCR rates were 16.6% for nondiabetics, 7.5% for diabetics not using metformin, and 35% for diabetics taking metformin, with metformin users having significantly higher pCR rates than either nondiabetics (*P* = 0.03) or diabetics not using metformin (*P* = 0.007). Metformin use was significantly associated with pCR rate on univariate (*P* = 0.05) and multivariate (*P* = 0.01) analyses. Furthermore, patients taking metformin had significantly increased disease-free (*P* = 0.013) and overall survival (*P* = 0.008) compared with other diabetic patients. Metformin use is associated with significantly higher pCR rates as well as improved survival. These promising data warrant further prospective study.

## Introduction

Rectal cancer is a common malignancy in the United States, with over 40,000 new diagnoses annually [1]. With the exception of early-stage rectal cancer, the primary mode of therapy for this disease in the curative setting involves preoperative chemoradiotherapy followed by a total mesorectal excision. The presence of pathologic complete response (pCR) following therapy is a powerful variable to determine the sensitivity of tumor to therapy, with pCR rates in rectal cancer around 15% in most studies [2,3]. Furthermore, pCR rate has been shown to be highly correlated with locoregional control (LRC), disease-free survival (DFS), and overall survival (OS) [2–6].

Multiple studies have been performed to examine clinical factors related to pCR rates in rectal cancer, including pretreatment carcinoembryonic antigen (CEA) level, circum-ferential extent, and distance from the anal verge (AV) among others [6]. However, patient-related factors also play a role, specifically the presence of diabetes mellitus. At least one study has found that diabetics have much lower pCR rates than nondiabetics (0% vs. 23%) [7]. Furthermore, several studies have shown that diabetics, as a group, are at great risk of developing rectal cancer and have poorer outcomes due to this diagnosis than nondiabetics [8–10]. The mechanism for this phenomenon remains unclear, ranging from increased levels of the promitogenic factor insulin to decreased chemotherapy delivery to the tumor due to altered microvasculature.

However, this poor response to therapy and its consequent effect on outcomes may not be present in all diabetics. In one large, population-based study, the use of metformin significantly decreased the risk of colorectal cancer [11]. This was also observed in a meta-analysis of available retrospective data [12]. One prospective clinical study found that the use of metformin was found to decrease the number of aberrant crypt foci (a surrogate marker for colorectal cancer risk) [13]. Furthermore, in retrospective review, diabetic patients taking metformin had improved OS compared with those not taking metformin after a diagnosis of colon cancer [14]. In other disease sites, metformin use has been associated with improved response to chemotherapy [15], as well as improved outcomes after treatment [16–18]. On the basis of these data, we wished to determine if pCR rate following neoadjuvant chemoradiation in a well-characterized cohort of patients treated at a single institution was affected by the use of metformin.

## Materials and Methods

### Patient and treatment characteristics

This study was approved by the University of Texas MD Anderson Institutional Review Board. The charts of 482 patients with rectal adenocarcinoma (≤12 cm from the AV) treated from 1996 to 2009 with neoadjuvant chemoradiation followed by surgical resection at a single institution were reviewed. Patients treated with surgery or radiation alone and those with distant metastatic disease at the time of presentation were excluded from the analysis. Prior to treatment, patients were evaluated by digital rectal examination, rigid proctoscopy, endoscopic ultrasound (or endorectal MRI), and computed tomography (CT) where appropriate. Staging was according to the American Joint Committee on Cancer TNM classification, 6th edition, and was completed using CT and endoscopic ultrasound. In this study, the majority of patients were T3 (86%), with similar number of patients presenting with N0 (40.5%) and N1 (55.4%) disease.

Patients were treated as described previously [5,6]. Surgical resection consisted of proctectomy with coloanal anastomosis, low anterior resection, or abdominoperineal resection. Radiotherapy was delivered to the primary site as well as the pelvic nodal beds using 3D-conformal technique. Chemotherapy was delivered concurrently and generally consisted of either 5-fluorouracil (5-FU, 53%) or capecitabine (45%). Pathologic response was graded in the surgical specimen by the pathologist at the time of surgical resection. pCR was defined as an absence of tumor cells in the primary specimen and sampled lymph nodes. Adjuvant chemotherapy was given to the majority of patients (81%) with the remaining patients either declining adjuvant therapy secondary to patient preference or inability to tolerate further systemic therapy.

Patients were categorized as: nondiabetic (*N* = 422), diabetic not taking metformin (*N* = 40), or diabetic taking metformin (*N* = 20). Patients were classified as diabetic due to either a preexisting diagnosis of diabetes upon presentation or a new diagnosis of diabetes prior to chemoradiation. All diabetic medications being taken by the patient at the start of therapy were obtained from the patient's chart or pharmacy record. Patients were then stratified according to metformin use, alone or in combination with other diabetic medications. Pretreatment height and weight were used to generate a pretreatment body mass index (BMI) when available (85.4%). Nonfasting blood glucose values were recorded within 1 month prior to treatment for most patients (87%).

### Statistical analysis

The primary outcome of this study was pCR, with secondary outcomes including OS, DFS, LRC, and time to distant metastasis (DM). All survival outcomes were dated from the time of diagnosis. OS was defined as the total survival time from diagnosis. DFS was defined as the time to the development of any recurrence or death. LRC was defined as the time to recurrence of disease within the primary site or regional lymph nodes. Time to DM was defined as the time to recurrence of disease at distant sites or nonregional lymph nodes. Univariate analysis of pCR rates was performed using logistic regression, with the following variables included: metformin use category, pretreatment nonfasting blood glucose levels, insulin use, age, BMI, tumor classification, nodal classification, tumor distance from the AV, circumferential tumor extent, pretreatment CEA, and tumor size. Any variable with a significance of *P* ≤ 0.1 was included in the multivariate analysis. Survival curves were generated using the Kaplan–Meier method and were compared using log-rank statistics. All *P*-values are two sided, with a *P* < 0.05 considered significant.

## Results

### Characteristics of treatment groups

In general, the patients in each group (nondiabetic, diabetics not taking metformin, and diabetics taking metformin) had similar patient characteristics (Table 1). No significant differences were found between groups in gender, race, tumor or nodal stage, distance from the AV, circumferential extent, pretreatment CEA level, pathologic differentiation, concurrent chemotherapy, or radiotherapy dose (Tables 2 and 3). Tumors were slightly larger in the diabetics not taking metformin (median length 6 cm) compared with nondiabetics (5 cm) and diabetics taking metformin (4.5 cm) (*P* = 0.01). Furthermore, diabetic patients were slightly older and had a greater BMI compared with nondiabetics (*P* = 0.003 and *P* = 0.002 respectively). Nonfasting blood glucose levels were also higher in diabetics (median 125.5) compared with nondiabetics (median 97, *P* = 0.01).

**Table 1:** Patient characteristics

	All patients		Nondiabetic		Diabetic, metformin		Diabetic, no metformin		
	*N*	%	*N*	%	*N*	%	*N*	%	*P*
All patients	482	100.0%	422	100.0%	20	100.0%	40	100.0%	
Median age (range), years	58	(19–84)	57	(19–84)	62	(43–74)	63.4	(37–76)	0.003
Median body mass index (range)	27	(17–70)	27	(17–70)	31	(21–47)	31	(22–48)	0.002
Gender									
Male	308	63.9%	263	62.3%	16	80.0%	29	72.5%	0.14
Female	174	36.1%	159	37.7%	4	20.0%	11	27.5%	
Race									
African-American	25	5.2%	20	4.7%	1	5.0%	4	10.0%	0.64
Asian	23	4.8%	20	4.7%	2	10.0%	1	2.5%	
Caucasian	371	77.0%	328	77.7%	13	65.0%	30	75.0%	
Hispanic	52	10.8%	44	10.4%	4	20.0%	4	10.0%	
Other	11	2.3%	10	2.4%	0	0.0%	1	2.5%	
Insulin use									
No	473	98.1%			17	85.0%	34	85.0%	0.84
Yes	9	1.9%			3	15.0%	6	15.0%	
Median pre-Tx blood glucose (range)	98	(59–368)	97	(59–245)	125.5	(77–226)	126	(73–368)	0.01
Median F/U (months)	101	(0–206)	103	(4–206)	92	(0–183)	77	(0–206)	

**Table 2:** Disease characteristics

	All patients		Nondiabetic		Diabetic, metformin		Diabetic, no metformin		
	*N*	%	*N*	%	*N*	%	*N*	%	*P*
Clinical tumor stage									
T2	20	4.1%	18	4.3%	1	5.0%	1	2.5%	0.7
T3	414	85.9%	365	86.5%	17	85.0%	32	80.0%	
T4	44	9.1%	36	8.5%	2	10.0%	6	15.0%	
Clinical nodal stage									
N0	195	40.5%	170	40.3%	9	45.0%	16	40.0%	0.15
N1	267	55.4%	236	55.9%	9	45.0%	22	55.0%	
N2	10	2.1%	7	1.7%	2	10.0%	1	2.5%	
Median tumor size (range), cm	5	(1–15)	5	(1–15)	4.5	(2–8)	6	(1.5–12)	0.01
Median distance from AV (range), cm	5.5	(0–14)	5.5	(0–14)	7	(0–11)	5	(0–10)	0.41
Median circumferential extent (range), %	50	(15–100)	50	(15–100)	75	(40–100)	60	(25–100)	0.16
Median pretreatment CEA (range), ng/mL	2.2	(0.4–185)	2.15	(0.4–185)	2.7	(0.5–108)	3.6	(0.5–30.2)	0.12
Differentiation									
Well	21	4.4%	17	4.0%	1	5.0%	3	7.5%	0.83
Moderate	391	81.1%	345	81.8%	16	80.0%	30	75.0%	
Poor	32	6.6%	29	6.9%	1	5.0%	2	5.0%	
Unknown	38	7.9%	31	7.3%	2	10.0%	5	12.5%	

AV, anal verge; CEA, carcinoembryonic antigen.

**Table 3:** Treatment characteristics

	All patients		Nondiabetic		Diabetic, metformin		Diabetic, no metformin		
	*N*	%	*N*	%	*N*	%	*N*	%	*P*
Median XRT dose (range), Gy	50.4	(20–63)	50.4	(20–63)	50.4	(45–52.5)	50.4	(45–52.5)	0.72
Concurrent chemotherapy									
Capecitabine	217	45.0%	188	44.5%	14	70.0%	15	37.5%	0.4
5-FU	255	52.9%	225	53.3%	6	30.0%	24	60.0%	
Uracil/tegafur	9	1.9%	8	1.9%	0	0.0%	1	2.5%	
Other	1	0.2%	1	0.2%	0	0.0%	0	0.0%	
Adjuvant chemotherapy									
Yes	392	81.3%	349	82.7%	16	80.0%	27	67.5%	0.33
No	76	15.8%	64	15.2%	3	15.0%	9	22.5%	

XRT, radiotherapy; 5-FU, 5-flurouracil.

### Pathologic response

Of the 482 patients, 80 (17%) were found to have a pCR at the time of surgical resection. On univariate analysis of the patient population, metformin treatment category was associated with increased rates of pCR (*P* = 0.03, Table 4). Also significant on univariate analysis of pCR rate were circumferential extent (*P* = 0.05) and pretreatment CEA (*P* = 0.01). BMI (*P* = 0.08) and tumor classification (*P* = 0.07) trended to a significant association with pCR and were included in the multivariate analysis. After accounting for the above factors on multivariate analysis, only metformin use (*P* = 0.01) and pretreatment CEA (*P* = 0.05) remained significantly associated with pCR (Table 5).

**Table 4:** Univariate analysis of pathologic complete response rates

UVA	Comparison	Sig.
Diabetic category		0.05
	Diabetic, metformin use vs. no metformin	0.02
	Diabetic, metformin use vs. nondiabetic	0.02
	Nondiabetic vs. diabetic, no metformin	0.05
Pre-Tx blood glucose	Continuous	0.6
Insulin use	Insulin vs. no insulin	0.2
Age	Continuous	0.2
Body mass index	≥30 vs. <30	0.08
Clinical tumor stage	4 vs. 2–3	0.07
Clinical nodal stage	1–2 vs. 0	0.57
Distance from anal verge	≥6 cm vs. <6 cm	0.44
Circumferential Invasion	≥50% vs. <50%	0.05
Pretreatment CEA	≥2.2 ng/dL vs. <2.2 ng/dL	0.01
Size	≥5.5 cm vs. <5.5 cm	0.13

UVA, univariate analysis.

**Table 5:** Multivariate analysis of pathologic complete response rates

MVA	Comparison	Sig.	OR	95% CI	
				Lower	Upper
Metformin category		0.01			
	Diabetic, metformin use vs. no metformin	0.02	16.8	1.6	181.1
	Diabetic, metformin use vs. nondiabetic	3 × 10^−3^	6.3	1.9	21.4
	Nondiabetic vs. diabetic, no metformin	0.36	2.7	0.3	22.1
Body mass index	≥30 vs. <30	0.43	0.8	0.4	1.5
Clinical tumor stage	4 vs. 2–3	1		0	
Circumferential invasion (%)	≥50% vs. <50%	0.1	0.5	0.3	1.1
Pretreatment CEA	≥2.2 ng/dL vs. <2.2 ng/dL	0.05	0.5	0.2	1.0

MVA, multivariate analysis; OR, odds ratio; 95% CI, 95% confidence interval.

**Figure 1:**
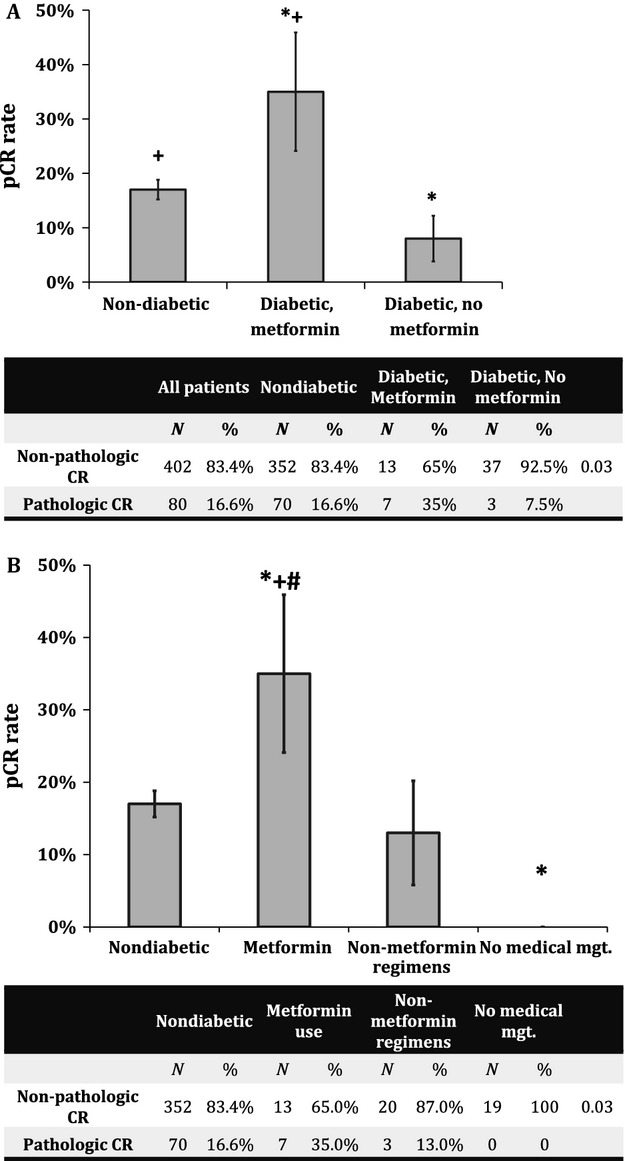
Pathologic complete response (pCR) rates following chemoradiotherapy. (A) pCR rates among the different metformin use categories. *Significantly different from nondiabetic patients. +Significantly different from diabetic patients not taking metformin. (B) pCR rates stratified by hypoglycemic use. *Significantly different from nondiabetic patients. +Significantly different from patients using nonmetformin hypoglycemic, #Significantly different from patients treated with no medical management for their diabetes. *P*-values <0.05 were considered significant. Error bars denote standard error of the mean.

Specifically, pCR rate in diabetics taking metformin was 35% (seven patients of 20) compared with 16.6% (70 patients of 422) in nondiabetics and 7.5% (three patients of 40) in diabetics with no medical management or managing their condition via other hypoglycemics. The overall interaction of the metformin treatment group and pCR was significant on analyses of variance (ANOVA) (Fig. 1A, *P* = 0.03). Furthermore, post hoc comparisons revealed that patients taking metformin had a significantly higher rate of pCR than either nondiabetics (*P* = 0.03) or diabetics not taking metformin (*P* = 0.007). After further stratification of diabetic patients by medical management, patients taking metformin continued to have a significantly improved pCR rate compared with those patients using other hypoglycemics (15%, *P* = 0.05) or those with no medical management of their diabetes (0%, *P* = 0.003) (Fig. 1B).

### Overall survival

**Figure 2:**
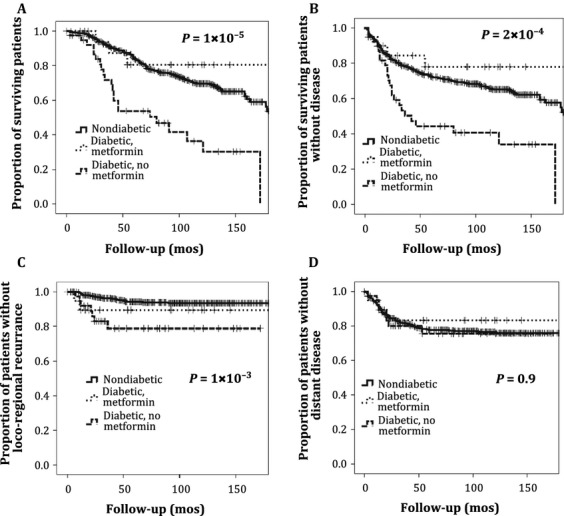
Survival outcomes and patterns of failure. (A) Overall survival, (B) disease-free survival, (C) locoregional control, and (D) time to distant metastasis.

The 5- and 10-year OS rates for the study population were 82% and 67%, respectively. In general, metformin use category was significantly associated with OS (*P* = 1 × 10^−5^, Fig. 2A). Patients taking metformin had significantly increased 5- and 10-year OS rates (81% and 79%) compared with diabetics not taking metformin (56% and 39%, *P* = 0.022) (Fig. 2A). Nondiabetics also had significantly higher 5- and 10-year OS rates (85% and 69%) than diabetics not taking metformin (*P* = 3 × 10^−7^). The OS rates were similar between nondiabetics and diabetics taking metformin (*P* = 0.8). On further analysis, diabetics taking metformin had significantly higher 5- and 10-year OS rates than either diabetics using other forms of hypoglycemic agents (52% and 41%, *P* = 0.012) or those diabetics with no medical management (65% and 42%, *P* = 0.025). No significant difference in OS was observed among diabetic patients taking insulin compared with those who were not (*P* = 0.83).

### Disease-free survival

Disease free survival rates at 5 and 10 years for the study population were 75% and 65%, respectively. Metformin use category was a significant predictor of DFS rate (*P* = 2 × 10^−4^, Fig. 2B). Similar to OS rates, patients taking metformin had significantly higher 5- and 10-year DFS rates (81% and 79%) compared with diabetics not taking metformin (41% and 39%, *P* = 0.01). In addition, nondiabetics had higher 5- and 10-year DFS rates (77% and 67%) than diabetics not taking metformin (*P* = 9 × 10^−5^). No significant difference in 5- and 10-year DFS rates between nondiabetics and diabetics taking metformin was observed (*P* = 0.56). On analysis stratified by diabetic control regimen, patients taking metformin had significantly improved 5- and 10-year DFS rates compared with patients using other hypoglycemic agents (44% and 37%, *P* = 0.017) or no medical management (53% and 42%, *P* = 0.04). No significant difference in DFS was observed between diabetic patients using insulin for glucose control and those who did not (*P* = 0.72).

### Locoregional and distant recurrence

The 5- and 10-year LRC rates for the study population were 92% and 90%, respectively. Metformin treatment category was significantly associated with LRC (*P* = 1 × 10^−3^). Specifically, nondiabetics had significantly higher LRC rates at 5 and 10 years than diabetics (*P* = 2 × 10^−4^), with no significant difference in LRC rates between nondiabetics and diabetics taking metformin (*P* = 0.3). Diabetics taking metformin had an increased rate of LRC compared with other diabetic patients; however, this was not statistically significant (*P* = 0.15). The 5- and 10-year time to DM rates for the study population were 77% and 71%, respectively. There was no significant association between metformin category and DM rate (*P* = 0.9).

## Discussion

The intersection between diabetes and rectal cancer is well known, with several epidemiologic and retrospective studies showing a relationship between diabetes and the development of rectal cancer as well as poorer overall outcome [8–10]. Furthermore, at least one study has shown decreased pCR rate in rectal cancer following chemoradiotherapy in diabetics [7]. However, the mechanism behind this phenomenon remains unclear. In this study, we found that pCR rate in diabetics using metformin for glycemic control (35%) was significantly greater than that of diabetics not taking metformin (7.5%) and nondiabetics (17%). The use of metformin remained significantly associated with increased pCR rate after controlling for significant factors on univariate analysis, including obesity. Finally, both DFS and OS rates were higher in patients taking metformin, with a trend to improved LRC. First, these data seem to suggest that the diabetic phenotype with respect to rectal cancer can be reversed via the use of metformin. Second, as pCR rates in diabetics taking metformin were significantly elevated compared with nondiabetics, metformin may function as a radiosensitizer beyond its role in affecting the patient's diabetic state.

These observations could be due to a number of factors. Most type II diabetics have elevated insulin and insulin-like growth factor (IGF)-1 levels at baseline, which are mitogenic to tumor cells via activation of downstream signaling cascades that mediate proliferation, migration/invasion, angiogenesis, and treatment resistance. Furthermore, elevated blood levels of these hormones have been related to the development of colorectal cancer [19]. Finally, several studies have found an association between obesity and the metabolic syndrome and both the development and outcome due to colorectal cancer [20–22], although this relationship is not always apparent [23,24]. One possibility is that metformin exerts its antineoplastic effects via normalization of circulating levels of IGF-1 and insulin. At least one study has shown that prediagnosis levels of insulin precursors are associated with decreased survival in colorectal cancer; however, this was not found to be true for levels of IGF-1 [25]. Furthermore, this hypothesis is supported by a recent placebo-controlled, randomized trial examining the use of metformin alone in the neoadjuvant setting in breast cancer [26]. In this study, metformin was found to have an antiproliferative effect on tumors, but only in women with insulin resistance, implying that metformin's primary effect in breast cancer was indirect via normalization of the tumor milieu.

However, in this study, we found significantly higher rates of pCR in patients taking metformin than those observed in nondiabetics, a finding similar to that seen by Jiralerspong et al. [15]. In their study, a trend to improved pCR was noted in diabetic breast cancer patients taking metformin and treated with chemotherapy compared with nondiabetic patients. Also, in this study, we noted that diabetic patients taking nonmetformin containing regimens (as opposed to diet-controlled diabetics) had similar pCR rates compared with nondiabetics. However, pCR rates in both groups were significantly less than those observed in the metformin group. Thus, metformin may have a direct effect on tumor cells independent of its effects on circulating insulin and IGF-1. One known target of metformin is the AMP-activated protein kinase (AMPK). This kinase is a component of the cellular energy sensing mechanism and is known to be critical for the effect of metformin on hepatic gluconeogenesis. Activation of AMPK inhibits the mammalian target of rapamycin (mTOR), a protein known to be a driver of cellular survival and proliferation in human cancer and one that is activated by IGF-1 [27]. In *in vitro* and preclinical models, metformin has been shown to activate AMPK and inhibit mTOR, leading to decreased cellular proliferation [28,29] and increased sensitivity to therapy [18, 29–33]. In colorectal cancer, activated AMPK, has been shown to be associated with improved disease-specific survival, providing a link between metformin, AMPK and improved outcome in this disease [34].

Although significant data exist linking metformin use and improved outcome, the clinical data are primarily retrospective and this study is no exception. The number of patients found to be taking metformin was limited in this study (20 patients) and the compliance to the use of metformin was not available in this study. Furthermore, while pretreatment glucose levels were similar between diabetic groups, hemoglobin A1C levels were not available in this patient population. Although the different treatment groups were well balanced regarding most factors known to relate to both pCR and survival outcomes, this does not completely eliminate the possibility of selection bias in patients taking metformin. However, we did observe a DFS and OS benefit to metformin treatment, which reflects favorably upon metformin as a possible adjuvant to current therapies as well as pCR rate as a reasonable surrogate endpoint for this treatment. Furthermore, metformin use appears to primarily affect LRC as opposed to DM. This seems to be consistent with an effect on the radiotherapy component of concurrent chemoradiation and is consistent with a radiosensitization effect of metformin observed in other disease sites [18, 30, 35].

In summary, in this study, we show an association between metformin use and improved tumor response to neoadjuvant chemoradiation in rectal cancer as well as improved survival outcomes. This provides a strong rationale for further prospective study of the use of metformin as an adjunct to the current standard of care in the treatment of locally advanced rectal cancer.

## References

[1] Jemal A., Siegel R., Xu J., Ward E. (2010). Cancer statistics. CA Cancer J. Clin..

[2] Maas M., Nelemans P. J., Valentini V., Das P., Rödel C., Kuo L-J. (2010). Long-term outcome in patients with a pathological complete response after chemoradiation for rectal cancer: a pooled analysis of individual patient data. Lancet Oncol..

[3] Zorcolo L., Rosman A. S., Restivo A., Pisano M., Nigri G. R., Fancellu A. (2012). Complete pathologic response after combined modality treatment for rectal cancer and long-term survival: a meta-analysis. Ann. Surg. Oncol..

[4] Kong M., Hong S. E., Choi W. S., Kim S.-Y., Choi J. (2012). Preoperative concurrent chemoradiotherapy for locally advanced rectal cancer: treatment outcomes and analysis of prognostic factors. Cancer Res. Treat..

[5] Park I. J., You Y. N., Agarwal A., Skibber J. M., Rodriguez-Bigas M. A., Eng C. (2012). Neoadjuvant treatment response as an early response indicator for patients with rectal cancer. J. Clin. Oncol..

[6] Das P., Skibber J. M., Rodriguez-Bigas M. A., Feig B. W., Chang G. J., Wolff R. A. (2007). Predictors of tumor response and downstaging in patients who receive preoperative chemoradiation for rectal cancer. Cancer.

[7] Caudle A. S., Kim H. J., Tepper J. E., O'Neil B. H., Lange L. A., Goldberg R. M. (2008). Diabetes mellitus affects response to neoadjuvant chemoradiotherapy in the management of rectal cancer. Ann. Surg. Oncol..

[8] Van de Poll-Franse L. V., Haak H. R., Coebergh J. W. W., Janssen-Heijnen M. L. G., Lemmens V. E. P. P. (2012). Disease-specific mortality among stage I–III colorectal cancer patients with diabetes: a large population-based analysis. Diabetologia.

[9] Dehal A. N., Newton C. C., Jacobs E. J., Patel A. V., Gapstur S. M., Campbell P. T. (2012). Impact of diabetes mellitus and insulin use on survival after colorectal cancer diagnosis: the Cancer Prevention Study-II Nutrition Cohort. J. Clin. Oncol..

[10] Coughlin S. S., Calle E. E., Teras L. R., Petrelli J., Thun M. J. (2004). Diabetes mellitus as a predictor of cancer mortality in a large cohort of US adults. Am. J. Epidemiol..

[11] Tseng C.-H. (2012). Diabetes, metformin use, and colon cancer: a population-based cohort study in Taiwan. Eur. J. Endocrinol..

[12] Soranna D., Scotti L., Zambon A., Bosetti C., Grassi G., Catapano A. (2012). Cancer risk associated with use of metformin and sulfonylurea in type 2 diabetes: a meta-analysis. Oncologist.

[13] Hosono K., Endo H., Takahashi H., Sugiyama M., Sakai E., Uchiyama T. (2010). Metformin suppresses colorectal aberrant crypt foci in a short-term clinical trial. Cancer Prev. Res. (Phila.).

[14] Garrett C. R., Hassabo H. M., Bhadkamkar N. A., Wen S., Baladandayuthapani V., Kee B. K. (2012). Survival advantage observed with the use of metformin in patients with type II diabetes and colorectal cancer. Br. J. Cancer.

[15] Jiralerspong S., Palla S. L., Giordano S. H., Meric-Bernstam F., Liedtke C., Barnett C. M. (2009). Metformin and pathologic complete responses to neoadjuvant chemotherapy in diabetic patients with breast cancer. J. Clin. Oncol..

[16] Sadeghi N., Abbruzzese J. L., Yeung S.-C. J., Hassan M., Li D. (2012). Metformin use is associated with better survival of diabetic patients with pancreatic cancer. Clin. Cancer Res..

[17] Currie C. J., Poole C. D., Jenkins-Jones S., Gale E. A. M., Johnson J. A., Morgan C. L. (2012). Mortality after incident cancer in people with and without type 2 diabetes: impact of metformin on survival. Diabetes Care.

[18] Skinner H. D., Sandulache V. C., Ow T. J., Meyn R. E., Yordy J. S., Beadle B. M. (2012). TP53 disruptive mutations lead to head and neck cancer treatment failure through inhibition of radiation-induced senescence. Clin. Cancer Res..

[19] Giovannucci E. (2001). Insulin, insulin-like growth factors and colon cancer: a review of the evidence. J. Nutr..

[20] Campbell P. T., Newton C. C., Dehal A. N., Jacobs E. J., Patel A. V., Gapstur S. M. (2012). Impact of body mass index on survival after colorectal cancer diagnosis: the Cancer Prevention Study-II Nutrition Cohort. J. Clin. Oncol..

[21] Meyerhardt J. A., Tepper J. E., Niedzwiecki D., Hollis D. R., McCollum A. D., Brady D. (2004). Impact of body mass index on outcomes and treatment-related toxicity in patients with stage II and III rectal cancer: findings from Intergroup Trial 0114. J. Clin. Oncol..

[22] Meyerhardt J. A., Catalano P. J., Haller D. G., Mayer R. J., Macdonald J. S., Benson A. B. (2003). Impact of diabetes mellitus on outcomes in patients with colon cancer. J. Clin. Oncol..

[23] Meyerhardt J. A., Niedzwiecki D., Hollis D., Saltz L. B., Mayer R. J., Nelson H. (2008). Impact of body mass index and weight change after treatment on cancer recurrence and survival in patients with stage III colon cancer: findings from cancer and leukemia Group B 89803. J. Clin. Oncol..

[24] Meyerhardt J. A., Ma J., Courneya K. S. (2010). Energetics in colorectal and prostate cancer. J. Clin. Oncol..

[25] Wolpin B. M., Meyerhardt J. A., Chan A. T., Ng K., Chan J. A., Wu K. (2009). Insulin, the insulin-like growth factor axis, and mortality in patients with nonmetastatic colorectal cancer. J. Clin. Oncol..

[26] Bonanni B., Puntoni M., Cazzaniga M., Pruneri G., Serrano D., Guerrieri-Gonzaga A. (2012). Dual effect of metformin on breast cancer proliferation in a randomized presurgical trial. J. Clin. Oncol..

[27] Feng Z. (2010). p53 regulation of the IGF-1/AKT/mTOR pathways and the endosomal compartment. Cold Spring Harb. Perspect. Biol..

[28] Jalving M., Gietema J. A., Lefrandt J. D., De Jong S., Reyners A. K. L., Gans R. O. B. (2010). Metformin: taking away the candy for cancer?. Eur. J. Cancer.

[29] Pollak M. N. (2012). Investigating metformin for cancer prevention and treatment: the end of the beginning. Cancer Discov..

[30] Sandulache V. C., Skinner H. D., Ow T. J., Zhang A., Xia X., Luchak J. M. (2012). Individualizing antimetabolic treatment strategies for head and neck squamous cell carcinoma based on TP53 mutational status. Cancer.

[31] Buzzai M., Jones R. G., Amaravadi R. K., Lum J. J., DeBerardinis R. J., Zhao F. (2007). Systemic treatment with the antidiabetic drug metformin selectively impairs p53-deficient tumor cell growth. Cancer Res..

[32] Zou M.-H., Kirkpatrick S. S., Davis B. J., Nelson J. S., Wiles 4th W. G., Schlattner U. (2004). Activation of the AMP-activated protein kinase by the anti-diabetic drug metformin in vivo. Role of mitochondrial reactive nitrogen species. J. Biol. Chem..

[33] Rocha G. Z., Dias M. M., Ropelle E. R., Osório-Costa F., Rossato F. A., Vercesi A. E. (2011). Metformin amplifies chemotherapy-induced AMPK activation and antitumoral growth. Clin. Cancer Res..

[34] Baba Y., Nosho K., Shima K., Meyerhardt J. A., Chan A. T., Engelman J. A. (2010). Prognostic significance of AMP-activated protein kinase expression and modifying effect of MAPK3/1 in colorectal cancer. Br. J. Cancer.

[35] Sanli T., Rashid A., Liu C., Harding S., Bristow R. G., Cutz J-C. (2010). Ionizing radiation activates AMP-activated kinase (AMPK): a target for radiosensitization of human cancer cells. Int. J. Radiat. Oncol. Biol. Phys..

